# Baculovirus as an Ideal Radionuclide Reporter Gene Vector: A New Strategy for Monitoring the Fate of Human Stem Cells *In Vivo*

**DOI:** 10.1371/journal.pone.0061305

**Published:** 2013-04-15

**Authors:** Yu Pan, Shuai Liu, Haifei Wu, Jing Lv, Xiaoqian Xu, Yifan Zhang

**Affiliations:** 1 Department of Nuclear Medicine, Ruijin Hospital, School of Medicine, Shanghai Jiao Tong University, Shanghai, China; 2 Department of Hematology, Changhai Hospital, Secondary Military Medical University, Shanghai, China; University of Tampere, Finland

## Abstract

**Purpose:**

Radionuclide reporter gene imaging holds promise for non-invasive monitoring of transplanted stem cells. Thus, the feasibility of utilizing recombinant baculoviruses carrying the sodium iodide symporter (NIS) reporter gene in monitoring stem cell therapy by radionuclide imaging was explored in this study.

**Methods:**

Recombinant baculoviruses carrying NIS and green fluorescent protein (GFP) reporter genes (Bac-NIS and Bac-GFP) were constructed and used to infect human induced pluripotent stem cells (hiPSCs), human embryonic stem cells (hESCs) and human umbilical cord blood mesenchymal stem cells (hUCB-MSCs). Infection efficiency, total fluorescence intensity and duration of transgene expression were determined by flow cytometry. Cytotoxicity/proliferative effects of baculovirus on hUCB-MSCs were assessed using CCK-8 assays. ^125^I uptake and perchlorate inhibition assays were performed on Bac-NIS-infected hUCB-MSCs. Radionuclide imaging of mice transplanted with Bac-NIS-infected hUCB-MSCs was performed by NanoSPECT/CT imaging.

**Results:**

Infection efficiencies of recombinant baculovirus in hESCs, hiPSCs and hUCB-MSCs increased with increasing MOIs (27.3%, 35.8% and 95.6%, respectively, at MOI = 800). Almost no cytotoxicity and only slight effects on hUCB-MSCs proliferation were observed. Obvious GFP expression (40.6%) remained at 8 days post-infection. The radioiodide was functionally accumulated by NIS gene products and specifically inhibited by perchlorate (ClO_4_^-^). Radioiodide uptake, peaking at 30 min and gradually decreasing over time, significantly correlated with hUCB-MSCs cell number (*R^2^* = 0.994). Finally, radionuclide imaging showed Bac-NIS-infected hUCB-MSCs effectively accumulated radioiodide *in vivo*, which gradually weakened over time.

**Conclusion:**

Baculovirus as transgenic vector of radionuclide reporter gene imaging technology is a promising strategy for monitoring stem cell transplantation therapy.

## Introduction

Stem cells are important in basic research as well as clinical applications in cell therapy and drug screening due to their capabilities of self-renewal and multilineage differentiation. In recent years, human stem cell transplantation therapy has been explored in clinical trials for numerous degenerative diseases, such as cardiovascular system diseases [1], nerve system diseases [2], [3] and diabetes [4], [5], which have obtained significant achievements and shown prospects of wide applicability. There are many potential sources of human stem cells for therapy. The three main types are human embryonic stem cells (hESCs), human induced pluripotent stem cells (hiPSCs) and human somatic stem cells.

hESCs are capable of totipotent differentiation [6] and unlimited self-renewal [7]; however, some vital hurdles must be overcome. Aside from the ethical and political controversies associated with hESCs, issues such as potential immunogenicity [8], [9] and tumorigenicity [10], [11] are ever-present safety concerns. In 2006, hiPSCs were reported as a significant breakthrough and a major milestone in life science research [12], [13]. Unlike hESCs, the use of hiPSCs would avoid ethical and immunogenicity problems because they are derived from the patient’s autologous cells [14]. However, critical concerns and challenges remain with this promising stem cell technology [15], [16].

At present, the most clinically applicable stem cell type is human mesenchymal stem cells/mesenchymal stromal cells (hMSCs).Umbilical cord blood (UCB), which is readily available as it is usually discarded after the delivery of a baby, is an accepted source of hMSCs because hUCB-MSCs are less mature, less immunogenic and have a much higher capability of proliferation and expansion, compared to hMSCs from other sources [17]–[20]. More importantly, unrelated donor UCB units can be obtained and preserved in public or private banks for instant access. Recently, increasing number of preclinical studies using hUCB-MSCs as material for stem cell therapy have showed great potential in the treatment of human degenerative diseases [21]–[25]. Therefore, hUCB-MSCs may be a more practical material for stem cell transplantation therapy but will require further validation in long-term clinical trials.

Despite the potential of stem cell transplantation therapy as a novel and promising treatment, there are still numerous problems need to be solved [26], especially the ability to monitor the long-term fate of transplanted stem cells *in vivo* using non-invasive and sensitive methods, determining how stem cells integrate, proliferate and differentiate would be of great value for understanding their biology and for optimizing stem cell transplantation techniques to gain the maximum therapeutic benefits [27], [28].

Reporter gene imaging is a noninvasive, sensitive and repetitive method that has been developed rapidly in recent years for monitoring cells *in vivo*, especially the radionuclide reporter gene imaging system which has specific advantages of high sensitivity (<10^−9^ M) and specificity, quantitative measurement, and the ability to image multiple target sites and employ various biological tracers for functional assessment [29]–[31]. Moreover, the radionuclide reporter gene imaging also overcomes the shortages of other traditional imaging modalities (e.g. inability of optical imaging to detect cells deeper in the body and the inability of radionuclide labeling imaging or magnetic resonance imaging to distinguish viable cells from nonviable cells) [29].

In radionuclide reporter gene imaging systems, a transgenic vector is required for delivering the radionuclide reporter gene into target stem cells. Among the vectors (e.g., adenovirus, lentivirus, retrovirus and baculovirus) which have been commonly utilized for transgene delivery, baculovirus has several advantages compared with other vectors [32], [33] and have captured growing attention as a versatile and powerful vector system for production of proteins, vaccine development, *in vitro* and *in vivo* gene delivery, surface display of eukaryotic proteins, cell-based assays for drug development and cancer therapy [32], [34].

The sodium iodide symporter (NIS) can effectively participate in the uptake of radiounclides such as ^131^I (scintigraphic imaging), ^123^I (single photon emission computed tomography, SPECT), ^125^I (SPECT), ^124^I (positron emission tomography, PET) ^94m^TcO_4_^–^ (PET) and ^99m^TcO_4_^–^ (SPECT), and was considered as an excellent reporter gene for imaging [35]–[37]. Therefore, in this study, we infected hUCB-MSCs, hESCs and hiPSCs with a recombinant baculovirus carrying the GFP or NIS reporter gene to investigate the feasibility of baculovirus mediated radionuclide reporter gene imaging as a new strategy in monitoring human stem cells *in vivo*.

## Materials and Methods

### Construction and Preparation of Recombinant Baculovirus Vectors

The baculovirus vector pFBGFPR was a gift from the Institute of Molecular Biology (Hong Kong University, Hong Kong, China), and pcDNA-NIS was obtained from Sissy Jhiang (Ohio State University, Columbus, OH, USA). Recombinant baculovirus vectors carrying NIS or GFP reporter genes (Bac-NIS and Bac-GFP) were constructed and prepared as described previously [38]. These baculovirus vectors were stored in phosphate-buffered saline (PBS; pH 7.4) at 4°C, and the titer (pfu/ml) was determined by plaque assay.

### Stem Cell Cultures

hiPSCs and hESCs (X-01 cell line) were graciously provided by Lei Xiao [39], [40] (Institute of Biochemistry and Cell Biology, Chinese Academy of Sciences, Shanghai, China). hESCs were approved by the Howard Hughes Medical Institute (Harvard University, Boston, MA, USA; Harvard Agreement Number: A13080). All hESCs experiments were conducted in accordance with the guidelines for research on human embryonic stem cells, jointly issued by the Ministry of Science and Technology and the Ministry of Health of China, and approved by the Ethical Committee of Shanghai Institutes for Biological Sciences. hUCB-MSCs were supplied by the Hematology Department of Changhai Hospital (Secondary Military Medical University, Shanghai, China) after collection (the collection of tissue samples was approved by the Changhai Hospital Ethical Committee, and all patients provided written informed consent) and identification by cell-specific markers [CD14(−), CD34(−), CD106(−), CD45(−), HLA-DR(−), CD29(+), CD44(+), CD90(+), CD105(+), HLA-ABC(+)] and osteogenic and adipogenic differentiation (data not shown).

hiPSCs and hESCs were maintained in an undifferentiated, pluripotent state with 1000 IU/mL leukemia inhibitory factor (LIF; Millipore, Billerica, MA, USA) and grown over murine embryonic fibroblast feeder layers which had been inactivated by 10 µg/mL mitomycin C (Sigma, St. Louis, MO, USA). The stem cells were cultured in serum-free medium, which was composed of DMEM/F12 supplemented with 20% KnockOut Serum Replacement, 0.1 mM non-essential amino acids, 1 mM L-glutamine and 0.1 mM β-mercaptoethanol (all from Invitrogen, Carlsbad, CA, USA). The cells were passaged with collagenase type IV (Invitrogen) at a ratio of 1∶10 every 3 or 4 days. The culture conditions were determined as described by Amit *et al.* [41].

hUCB-MSCs were cultured in medium, which was composed of basal medium for human MSCs supplemented with hMSC stimulatory supplements (StemCell, Vancouver, BC, Canada), and routinely passaged with 0.05% trypsin-EDTA (Invitrogen) when reaching ∼80% confluency. Cells at the 5∼10th passages were used for the present study.

### Infection Efficiency of Stem Cells with Bac-GFP

hUCB-MSCs were seeded in 12-well plates at a density of 1×10^5^ cells per well. The medium was aspirated after 24 h, and cells were washed twice with PBS. Bac-GFP virus stock supernatant was adjusted with PBS to the final volume of 500 µl/well for different multiplicities of infection (MOI) of 0, 20, 50, 100, 200, 400, 600 or 800, then the hUCB-MSCs were incubated in above virus solution at 25∼27°C for 4 h, as described elsewhere [42]. The virus solution was aspirated after the infection, and cells were washed with PBS twice and replenished with fresh medium. The efficiency of GFP expression was observed using a fluorescent inverted phase contrast microscope after 24 h, and the percentage of GFP-positive cells (GFP^+^ %) and the mean fluorescence intensity (MFI) were analyzed by flow cytometry (blue light excitation wave-length: 488 nm; detection wave length: 520 nm) at the same time. The total fluorescence intensity (TFI) representing the total transgene expression level was calculated as follows: TFI = GFP^+^ %×MFI×cell number (100,000).

hiPSCs and hESCs were seeded in 12-well plates at a density of 1×10^5^ per well (cell density was determined after dissociating the cell clusters into single cells with trypsin-EDTA). The cell clusters were mechanically reduced to a size of about 200∼300 cells and replated onto Matrigel (BD Biosciences, Bedford, MA, USA) coated plates. The medium was aspirated after 24 h, and the cells were washed twice with PBS. Opti-MEM (Invitrogen) containing Bac-GFP at the MOI of 0, 50, 200 or 800 in a final volume of 500 µl was added to the cells and incubated at 25∼27°C for 4 h. Subsequently, the virus solution was aspirated, and cells were washed with PBS twice and replenished with fresh medium. The efficiency of GFP expression was observed using a fluorescent inverted phase contrast microscope after 24 h, and the GFP^+^ % and MFI were analyzed by flow cytometry at the same time after dissociating the cell clusters into single cells by trypsin-EDTA.

### Cell Viability and Proliferation Assays

hUCB-MSCs were seeded into 96-well plates at a density of 4×10^3^ cells per well. After 24 h, 100 µl PBS containing the virus supernatant at the MOI of 20, 50, 100, 200 or 400 was added into each well, respectively and incubated for 4 h. The control group was mock-infected hUCB-MSCs (MOI = 0), and the blank group contained only medium without cells. At 1, 3, 5, 7, 9, 11, 13 and 15 days post-infection (dpi), the Cell Counting Kit-8 reagent (CCK-8; Dojindo, Mashikimachi, kamimashiki gun Kumamoto, Japan) was added at 10 µl per well of each group after changing the medium with PBS, and the absorbance was read using a microplate spectrofluorometer (detection wavelength: 450 nm; reference wavelength: 650 nm). The cells were reseeded at 4×10^3^ per well when reaching ∼80% confluency.

Thereafter, the cell viability (%) was calculated using the following formula: cell viability (%) = (A_test_−A_blank_)/(A_control_−A_blank_)×100. Here, A_test_ represents absorbance of each experimental group, including Bac-NIS-infected cells, PBS and CCK-8 reagent; A_blank_ represents absorbance of the blank group, including only PBS and CCK-8 reagent; A_control_ represents absorbance of control group, including mock-infected cells, PBS and CCK-8 reagent.

### Durability of Bac-GFP Expression in Infected hUCB-MSCs

hUCB-MSCs were seeded in 12-well plates at a cell density of 1×10^5^ per well. After 24 h, the cells were infected with Bac-GFP virus at MOI = 200 (or mock-infected, MOI = 0) as above. The GFP^+^ % and MFI of infected hUCB-MSCs were analyzed by flow cytometry at 1, 2, 3, 4, 8, 12, 16 and 20 dpi until the GFP^+^ % was lower than 10%. Cell cultures were maintained by reseeding at 1×10^5^ per well when reaching ∼80% confluency.

### Iodide Uptake of Bac-NIS-infected hUCB-MSCs

hUCB-MSCs were seeded in 24-well plates at 2×10^4^ per well for 24 h. After washing the cells with PBS, 250 µl PBS containing the Bac-NIS virus supernatant at the MOI of 0, 20, 50, 100, 200, 400, 600 or 800 was added to each well for 4 h at 25∼27°C. Thereafter, the virus solution was removed, and cells were incubated for another 24 h at 37°C. Before the radioiodide uptake assay, the medium was aspirated, and cells were washed with 500 µl Hank’s balanced salt solution (HBSS), which was adjusted with Hepes buffer (Invitrogen) to pH 7.3. The iodide uptake assay was performed by adding 500 µl buffered HBSS containing 0.1 µCi (3.7 kBq) Na^125^I and 10 µM NaI to each well [43]. The cells were incubated at 37°C for 30 min, and then radioiodide uptake was terminated by aspirating the radioactive solution and washing the cells rapidly with 500 µl ice-cold buffered HBSS. To determine the intracellular amount of ^125^I, 500 µl 1 M NaOH was added to each well for 20 min, and then samples were transferred into vials for determination of counts per minute (cpm) with a γ counter. In dynamic iodide uptake assay, the cells in MOI = 0 and 200 groups were incubated with radioactive solution for various time points (5, 15, 30, 60, 90 and 120 min) before terminating the radioiodide uptake.

### NaClO_4_ Inhibition of Iodide Uptake

hUCB-MSCs were seeded into 24-well plates at 2×10^4^ per well for 24 h. The cells were then infected with Bac-NIS at MOI = 200. After infection, 500 µl buffered HBSS containing 0.1 µCi (3.7 kBq) Na^125^I, 10 µM NaI and various concentrations of NaClO_4_ (0 µM, 30 µM and 300 µM groups) were added. The cells were incubated at 37°C for 30 min, and then iodide uptake was terminated and determined with a γ counter as above.

### Correlation Analysis between ^125^I Uptake and Number of Bac-NIS-infected hUCB-MSCs *in vitro*

hUCB-MSCs were seeded in 24-well plates at densities of 5×10^3^, 1×10^4^, 2×10^4^, 4×10^4^ and 8×10^4^ per well for 24 h. The cells were then infected with Bac-NIS at MOI = 200 or 0 (as control group). The virus solution was removed after 4 h infection, and cells were incubated at 37°C for another 24 h. The radioiodide uptake assay of each group was then performed as above.

### Nano-single-photon Emission Computed Tomography/Computed Tomography (NanoSPECT/CT) Imaging of Nude Mice Transplanted with Bac-NIS-infected hUCB-MSCs

hUCB-MSCs were infected with Bac-NIS at MOI of 200. At 24 h post-infection (hpi), 1×10^7^ infected and mock-infected cells were respectively harvested, centrifuged, resuspended in 100 µl PBS and then transplanted subcutaneously into each axilla of male nude mice (BALB/c nu/nu, 4 weeks old) with a 1-inch needle. After administration with 300 µCi (11.1 MBq) Na^125^I via the tail vein, the mice were anesthetized with 4% isoflurane and then placed in a spread prone position on the scanner bed of NanoSPECT/CT (Bioscan, Washington, DC, USA). The mice were imaged at 30, 60 and 120 min under inhalational isoflurane anesthesia (1.5% in oxygen at flow rate of 0.6 L/min). NanoSPECT/CT images were reconstructed by using Bioscan InVivoScope® 1.43 software (Bioscan) and displayed in coronal, sagittal and horizontal planes for visual evaluation. All mice used in this study were purchased from Shanghai Slaccas Experiment Animal Corporation (Shanghai Institutes for Biological Science, Shanghai, China), and all procedures were conducted according to the Animal Care and Use guideline and were approved by the Institutional Animal Care and Use Committee of the Ruijin Hospital.

### Statistical Analysis

All data were processed using SAS ver. 8.02 software (SAS Institute Inc., Cary, NC, USA), and all graphs were drawn using GraphPad Prism ver. 5.01 software (GraphPad Software Inc., La Jolla, CA, USA). Data were expressed as means ± standard deviation (SD), and continuous variables were compared by the paired Student’s t-test. Differences between groups were compared by ANOVA using Dunnett’s Multiple Comparison Test. *P*-values less than 0.05 (two-tailed) were considered to be significant.

## Results

### Amplification and Determination of Recombinant Bac-GFP and Bac-NIS

The recombinant baculoviruses carrying GFP and NIS under the control of a cytomegalovirus immediate-early (CMV-IE) promoter were constructed successfully. The viruses were amplified, and titers determined by plaque assay reached as high as ∼1.4×10^9^ pfu/ml.

### Infection Efficiency of Bac-GFP in Human Stem Cells

**Figure 1:**
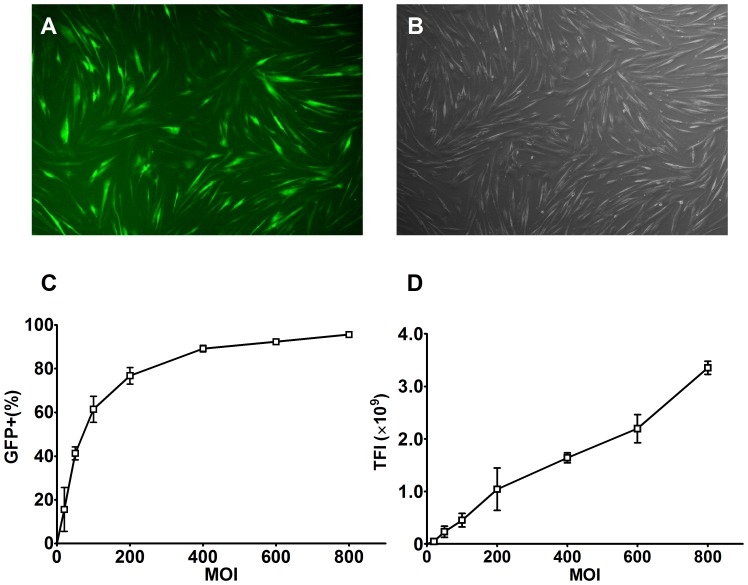
Infection efficiency of recombinant baculovirus in hUCB-MSCs. **A** and **B**: Fluorescence and bright field microscopy images of hUCB-MSCs, respectively, which were seeded at 1×10^5^ cells per well and infected with Bac-GFP at MOI = 200 (40×). **C** and **D**: GFP^+^ % and TFI of Bac-GFP-infected hUCB-MSCs at 24 hpi with different MOIs. Results are means ± SD (n = 3). **Abbreviations**: Bac-GFP, recombinant baculovirus carrying green fluorescence protein reporter gene; hUCB-MSCs, human umbilical cord blood mesenchymal stem cells; MOI, multiplicity of infection; GFP+%, percentage of GFP positive cells; TFI, total fluorescence intensity; SD, standard deviation.

The hUCB-MSCs with a fusiform shape were distributed uniformly at 24 h after infection with Bac-GFP, and the majority expressed GFP (Fig. 1A and B). The infection efficiency and total transgene expression level of infected hUCB-MSCs, expressed as GFP^+^ % (Fig. 1C) and TFI (Fig. 1D), respectively, were elevated with increasing MOI. The use of MOI = 800 yielded the highest GFP^+^ % and TFI, which were as high as 95.6% and 3.35×10^9^, respectively, while the GFP^+^ % reached 76.7% at the MOI of 200. Though the GFP^+^ % gradually approached saturation levels with the increase of MOI, the TFI still increased and seemed to positively correlate with the MOI. These findings clearly indicated that a higher infection efficiency and transgene expression level could be achieved by increasing MOI.

**Figure 2:**
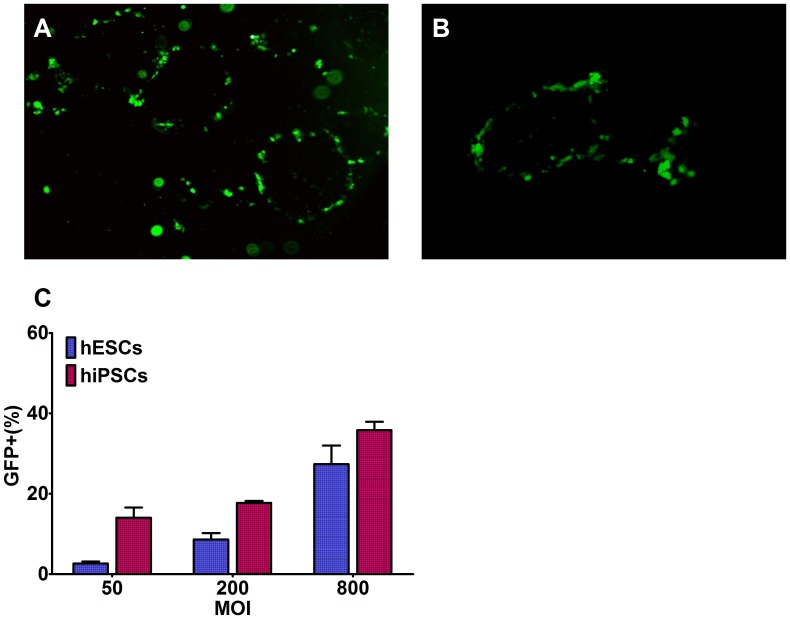
Infection efficiency of recombinant baculovirus in hESCs and hiPSCs. **A** and **B**: Fluorescence microscopy images of hESCs and hiPSCs, respectively, which were seeded at 1×10^5^ cells per well and infected with Bac-GFP at MOI = 200 (40×). **C**: GFP^+^ % of Bac-GFP-infected hESCs and hiPSCs at 24 hpi with different MOIs. Results are means ± SD (n = 3). **Abbreviations**: hESCs, human embryonic stem cells; hiPSCs, human induced pluripotent stem cells.

Observations by fluorescence microscopy revealed that the scattered cells and the cells surrounding the hESCs and hiPSCs cell clusters obviously expressed GFP, while little fluorescence was found in the center of the clusters (Fig. 2A and B). The GFP^+^ % of these two types stem cells were, respectively, 27.3% and 35.8% at MOI = 800, and only 8.6% and 17.7% at MOI = 200 (Fig. 2C).

### Effects of Recombinant Baculovirus on Viability and Proliferation of hUCB-MSCs

**Figure 3:**
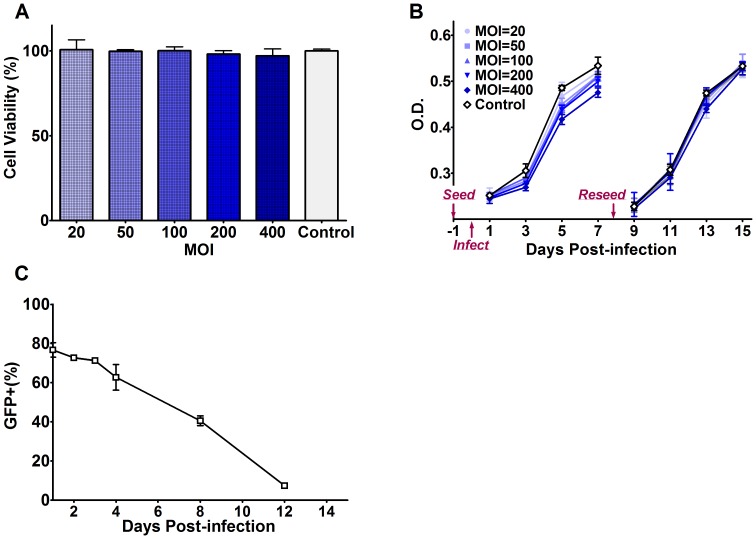
Effects and duration of transgene expression in baculovirus-infected hUCB-MSCs. **A**: Cell viability of hUCB-MSCs infected with Bac-NIS at different MOIs at 24 hpi as detected by the CCK-8 assay. **B**: Cell proliferation of hUCB-MSCs infected by Bac-NIS at different MOIs over time (1, 3, 5, 7, 9, 11, 13 and 15 dpi), as detected by the CCK-8 assay. The cells were seeded 1 day before infection with Bac-NIS and reseeded at 8 dpi when the cells reached ∼80% confluency. **C**: GFP^+^ % of Bac-GFP-infected hUCB-MSCs over time (1, 2, 3, 4, 8 and 12 dpi). The cells were reseeded at 8 dpi when reached ∼80% confluency. Results are means ± SD (n = 3). **Abbreviations**: Bac-NIS, recombinant baculovirus carrying sodium-iodide symporter reporter gene; dpi, days post-infection; CCK-8, Cell Counting Kit-8.

At 24 h after infection, cell viabilities of each MOI group were determined (Fig. 3A). There were no statistically significant differences between each experimental and control group. However, proliferation rates of infected cells were slightly lower than that of mock-infected cells, and the effects were more obvious with the increase of MOI. The proliferation rates of infected cells returned to normal levels and approached that of the mock-infected group after passaging (Fig. 3B).

### Durability of GFP Expression in Bac-GFP-infected hUCB-MSCs

As quantitatively determined by flow cytometry, the GFP expression of infected hUCB-MSCs decreased gradually over time after infection. The GFP^+^ % at 1, 2, 3, 4, 8 and 12 dpi were 76.7%, 72.7%, 71.2%, 62.7%, 40.6% and 7.4%, respectively (Fig. 3C), indicating that the Bac-GFP expression was especially stable in the first 4 days and remained at a high level for at least 1 week before declining with cell division.

### Radioiodide Uptake Assays of Bac-NIS-infected hUCB-MSCs *in vitro*

**Figure 4:**
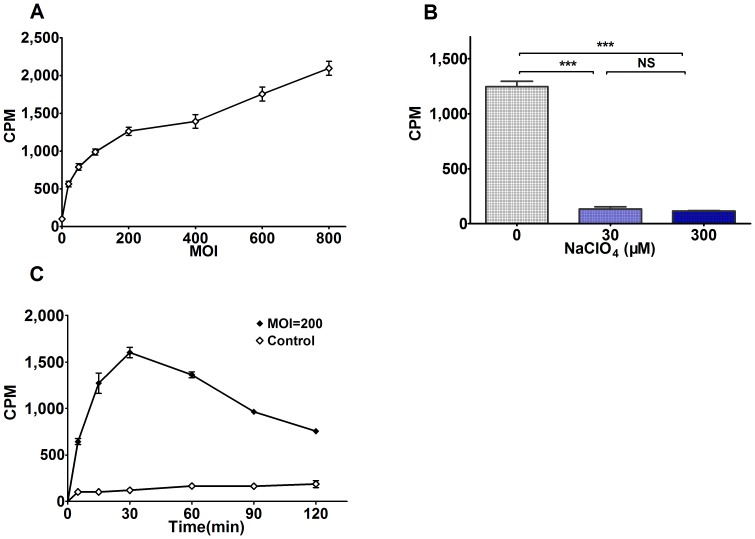
Radioiodide uptake of Bac-NIS-infected hUCB-MSCs *in vitro*. **A**: The cpm of intracellular ^125^I in Bac-NIS-infected hUCB-MSCs with different MOIs at 24 hpi. **B**: Inhibitory effects of NaClO_4_ on ^125^I uptake in Bac-NIS-infected hUCB-MSCs at MOI = 200. **C**: Dynamic ^125^I uptake curve of Bac-NIS-infected hUCB-MSCs at MOI = 200. Results are means ± SD (n = 3). **Abbreviations**: cpm, counts per minute; ***, P<0.001; NS, no statistically significant difference.

The amount of ^125^I uptake in Bac-NIS-infected hUCB-MSCs increased with increasing MOIs (Fig. 4A), and this was consistent with the TFI of Bac-GFP-infected cells at different MOIs, which also represented the total transgene expression level of a given number of cells (Fig. 1D).

Iodide uptake in thyroid cells can be specifically inhibited by ClO_4_^−^. In the iodide uptake inhibition assay, the ^125^I uptake of Bac-NIS-infected hUCB-MSCs was also inhibited by NaClO_4_ effectively (Fig. 4B). Even at the NaClO_4_ concentration of only 30 µM, the ^125^I uptake could be inhibited significantly. Compared with the 0 µM control group, the inhibition ratios of the NaClO_4_ 30 µM and 300 µM groups reached 89.3% and 90.9%, respectively, and there was no statistically significant difference between these two experimental groups.

In the dynamic radioiodide uptake assay, the amount of intracellular^ 125^I uptake increased dramatically within 30 min and peaked at 30 min. Thereafter, the level of intracellular ^125^I in Bac-NIS-infected hUCB-MSCs decreased gradually; meanwhile the ^125^I export increased. And there was no functional iodide uptake observed in the control group (Fig. 4C). These results also indicated that 30 min after administration with radioiodide may be the optimal time point for imaging of transplanted stem cells *in vivo*.

### Correlation Analysis between ^125^I Uptake and Number of Bac-NIS-infected hUCB-MSCs *in vitro*

**Figure 5:**
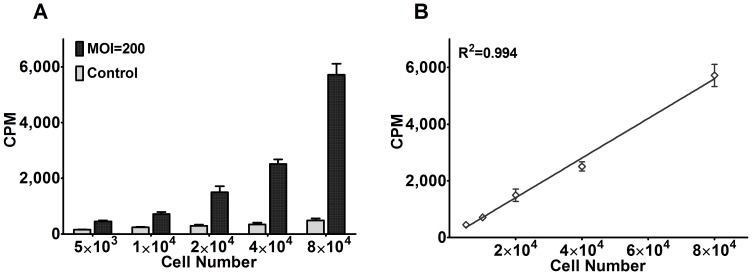
Correlation between ^125^I uptake in Bac-NIS-infected hUCB-MSCs and cell number *in vitro*. **A**: ^125^I uptake (cpm) in Bac-NIS-infected and mock-infected hUCB-MSCs at MOI = 200 in serially diluted cells (ranging from 5×10^3^ to 8×10^4^ per well). **B**: Correlation analysis between cpm and cell number of Bac-NIS-infected hUCB-MSCs. Results are means ± SD (n = 3).

To evaluate the correlation between the amount of ^125^I uptake and cell number of Bac-NIS-infected hUCB-MSCs, the cells were serially diluted from 5×10^3^ to 8×10^4^ per well. There was no statistically significant difference between each mock-infected group. By contrast, Bac-NIS-infected hUCB-MSCs showed a significant increase of ^125^I uptake along with increasing cell number (Fig. 5A). A rather high correlation (*R^2^* = 0.994, *P*<0.001) was observed between the ^125^I uptake amount and the cell number of infected cells (Fig. 5B), which provided a possibility of indirectly and quantitatively determining the viable cell number *in vivo* by measuring intracellular radionuclides.

### NanoSPECT/CT Imaging of Transplanted hUCB-MSCs *in vivo*

**Figure 6:**
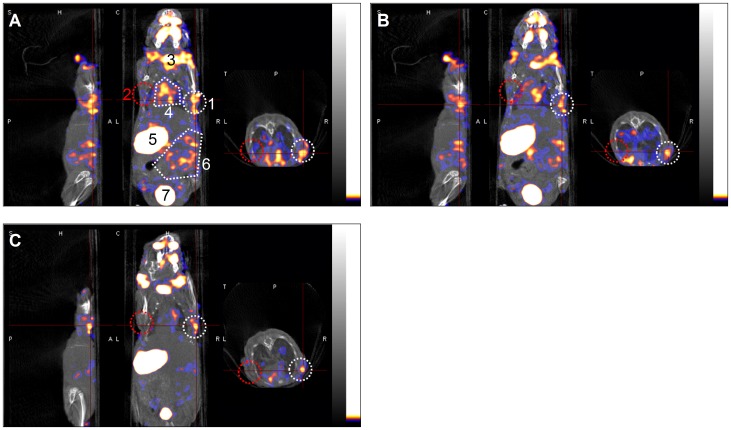
*In vivo* imaging of Bac-NIS-infected hUCB-MSCs transplantation with NanoSPECT/CT. **A**: Overlapping SPECT and CT images at 30 min after administration of 300 µCi (11.1 MBq) Na^125^I. The right axilla (white circular area) was transplanted with Bac-NIS-infected hUCB-MSCs (MOI = 200, 1×10^7^ cells) and showed a high radioiodide uptake, while the left axilla (red circular area) was transplanted with mock-infected cells as a control and showed no obvious radioiodide uptake. From left to right side are, respectively, the coronal, sagittal and horizontal sections. All CT images are shown with a grey palette, and all SPECT images are shown with a warm palette. **B** and **C**: SPECT/CT images at 60 min and 120 min after radioiodide administration. **Abbreviations**: CT, computed tomography; SPECT, single photon emission computed tomography; **1**, Bac-NIS-infected hUCB-MSCs; **2**, mock-infected hUCB-MSCs; **3**, thyroid; **4**, heart; **5**, stomach; **6**, intestinal area; **7**, bladder.

At 30 min after administration with Na^125^I, the right axilla of mice, where was transplanted with Bac-NIS-infected hUCB-MSCs (MOI = 200, 1×10^7^ cells) 24 h before, was obviously visible by SPECT imaging, whereas the left axilla transplanted with mock-infected hUCB-MSCs as a control was not. At the same time, radioactivity signals were observed in the pharynx oralis, salivary gland, thyroid, heart, stomach and intestines area. The bladder was also visible as a result of ^125^I excretion in the urine (Fig. 6A). By observation at 60 min and 120 min after administration with Na^125^I, radioactivity in the right axilla area appeared to decrease over time, although it was still clearly visible at 120 min. While radioactivity in the heart and intestines area gradually reduced and eventually vanished, the thyroid and stomach sustained high radioactivity levels, as did the bladder as the excretion pathway (Fig. 6B and C).

## Discussion

In recent years, molecular imaging based on radionuclide technology, which enable non-invasive, repetitive and quantitative visualization of various cellular events and exogenous/endogenous gene expression in living organisms, has been rapidly developed and widely used in the biomedical research field. The most widely used radionuclide reporter gene imaging strategy for monitoring and evaluating stem cell transplantation therapy currently is indirect method of using reporter genes and their “radionuclide reporter probes”. For this strategy, it is obvious that an ideal transgenic vector is crucially important for transducing the radionuclide reporter genes into target stem cells.

In this study, we constructed a recombinant baculovirus containing the CMV-IE promoter to transduce the GFP reporter gene into three types of stem cells. The recombinant baculovirus was found to infect hUCB-MSCs efficiently and reach a remarkable 76.7% at the MOI of 200 without assistance of any reagent like butyrate. However, the infection efficiencies in hESCs and hiPSCs were much lower (8.6% and 17.7% respectively) at MOI = 200, and improved but were still not ideal at MOI = 800 (27.3% and 35.8%, respectively). The main reason for this phenomenon may be due to the promoter of the recombinant baculovirus. Zeng *et al.* [44] and Du *et al.* [45] found that the woodchuck hepatitis post-transcriptional regulatory element (WPRE) and the human elongation factor 1-α (EF-1α) promoter could significantly enhance the baculovirus infection efficiency in hESCs. In addition, the formation and growth of stem cell clusters, the infection surrounding solution medium, and the infection time and temperature before virus removal have also been found to contribute to the infection efficiency [46]–[48]. Thus, improving the infection conditions for hESCs and hiPSCs with baculovirus vector will require further studies.

In order to address biosafety concerns, we assessed the effects of recombinant baculovirus on the viability and proliferation of hUCB-MSCs in this study. The recombinant baculovirus did not compromise the stem cell viability but somewhat impeded the cell proliferation rate, which could be restored to normal levels after passaging. These transient effects may be explained by the perturbation of host cell global gene expression and triggering of innate immune responses by the baculovirus [49]. Of note, baculoviruses are not known to either replicate inside stem cells or integrate its genome into host chromosomes in the absence of selective pressure [49]. It has also been reported that recombinant baculoviruses compromise neither the viability, phenotype and pluripotency/differentiation of stem cells [44], [50], nor the expression of human stem cell markers and teratoma formation [45].

Long-term transgene expression is also an important attribute of an ideal vector, therefore we examined the duration of reporter gene expression in baculovirus infected hUCB-MSCs and found that the transgene expression level remained at a relative high level for at least 8 dpi but nearly extinguished at 12 dpi. For baculovirus cannot replicate in mammalian cells, its viral genome and the transgene will dilute with cell division, which may be responsible for the gradual decrease of transgene expression. Recent studies on the development of baculoviruses for sustained long-term transgene expression may overcome this flaw and thus optimize this vector system for long-term monitoring of stem cells [51], [52].

This novel baculovirus transgenic vector has other advantageous features as well, such as neither replicating in mammalian cells nor randomly integrating into the host genome (randomly integration may lead to mutations) [53], [54]. It also has a packaging capacity of more than 100 kb [55], thus allowing for insertion of multiple genes and regulatory elements. Moreover, recombinant baculovirus construction is easy and can be propagated to high titers by infecting its natural host insect cells [56]. Furthermore, it can infect mammalian cells in the stationary phase as well as the mitotic phase. Taken above together, baculovirus may be a suitable transgenic vector of choice for infecting human stem cells in reporter gene imaging.

However, thus far, studies on radionuclide reporter gene imaging for hUCB-MSCs mediated by a baculovirus vector have not been reported. Therefore, in this study, we also successfully constructed and prepared a recombinant baculovirus carrying the NIS reporter gene for exploring the feasibility of utilizing the baculovirus as radionuclide reporter gene vector in monitoring hUCB-MSCs transplantation. The *in vitro* results showed that, the NIS proteins expressed in Bac-NIS-infected hUCB-MSCs allowed iodide uptake effectively, which could be specifically inhibited by perchlorate (ClO_4_^−^). The dynamic changes in radioiodide uptake, with a rapid increase and a slow decrease, provided a peak time point for monitoring transplanted hUCB-MSCs and also minimized radioactive damage on the stem cells. This study also demonstrated a significantly high correlation between the cell number of hUCB-MSCs and the level of radioiodide uptake, supporting the further possibility of this method for quantitatively monitoring transplanted stem cells *in vivo*.

In *in vivo* experiment, the hUCB-MSCs were infected with Bac-NIS *in vitro* before transplantation, which also avoided the limitation of baculovirus being inactivated by serum complement proteins, as would occur with the use of this vector directly *in vivo*. Then Bac-NIS-infected hUCB-MSCs were then transplanted subcutaneously into axillae of nude mice to preliminarily evaluate the feasibility of monitoring hUCB-MSCs *in vivo* by radionuclide reporter gene imaging. The Bac-NIS-infected hUCB-MSCs successfully expressed NIS proteins, which effectively mediated the accumulation of radioiodide *in vivo*, as detected by SPECT/CT, while no radioactivity was observed with mock-infected transplanted cells. The radioactivity of Bac-NIS-infected hUCB-MSCs *in* *vivo* was most obvious at 30 min after Na^125^I administration and gradually decreased over time. Therefore, imaging of transplanted hUCB-MSCs was determined to be most optimal at 30 min after radioiodide administration.

However, the radioactivity level of transplanted stem cells mainly depended on the transient radioiodide uptake from the flowing blood. The tissues expressing endogenous NIS (such as thyroid gland, salivary glands, stomach, lactating mammary glands, lachrymal glands, small intestine, rectum, heart and lung [57]–[59]), could accumulate radioiodide from the blood and then could be clearly observed in SPECT/CT imaging at an early period after administration (Fig. 6A). These non-specific radioiodide accumulations may interfere the imaging of transplanted stem cells or even lead to false results when the transplantation sites are located in or near these tissues, as would be the case, for example, if the stem cells are targeted for therapy of myocardial infarction. By contrast, stem cell transplantation in tissues such as liver, muscle or spinal cord, which have low endogenous NIS expression, will be ideal for NIS imaging. The feasible solution to this problem of endogenous NIS interference may be delaying imaging to a time point at which the intracellular radioiodide in these tissues is nearly eliminated, while transplanted cells in the same area are still clearly visible. Alternatively, the animal model can be pre-treated with thyroid blocking agents before stem cell transplantation, which can decrease the radioiodide uptake of thyroid and other tissues expressing endogenous NIS and thus increase the transient uptake of Bac-NIS-infected stem cells.

With rapid advances in human stem cell research and with high demands for testing their regenerative potential in preclinical models, we can foresee that non-invasive and repetitive stem cell *in vivo* imaging will play a critical role, and the strategy in our study of using baculovirus as a transgenic vector in radionuclide reporter gene imaging system may provide valuable data to support further studies of hUCB-MSCs (or other types of stem cells) transplantation therapy for specific diseases.

### Conclusion

In view of the results that have been obtained, this study showed that the baculovirus vector with significantly high transduction efficiency and low cytotoxicity is suitable and potential for transducing reporter genes into hUCB-MSCs *in vitro*. More importantly, this study also supported the feasibility of using the baculovirus vector expressing the NIS radionuclide reporter gene for non-invasively monitoring hUCB-MSCs transplantation therapy *in vivo*.
